# Trends of increase in western medical services in traditional medicine hospitals in china

**DOI:** 10.1186/1472-6963-11-212

**Published:** 2011-09-06

**Authors:** Jay J Shen, Ying Wang, Fang Lin, Jun Lu, Charles B Moseley, Mei Sun, Mo Hao

**Affiliations:** 1 Department of Health Care Administration and Policy, University of Nevada at Las Vegas, USA; 2 Research Institute of Health Development Strategies, Fudan University, China; 3 School of Business, University of Oklahoma, USA

**Keywords:** traditional Chinese medicine, Western medicine, hospital, revenue, health policy, China

## Abstract

**Background:**

Compare changes in types of hospital service revenues between traditional Chinese medicine (TCM) hospitals and Western-medicine based general hospitals.

**Methods:**

97 TCM hospitals and 103 general hospitals were surveyed in years of 2000 and 2004. Six types of medical service revenue between the two types of hospitals were compared overtime. The national statistics from 1999 to 2008 were also used as complementary evidence.

**Results:**

For TCM hospitals, the percentage of service revenue from Western medicine increased from 44.3% to 47.4% while the percentage of service revenue from TCM declined from 26.4% to 18.8% from 1999 to 2004. Percentages of revenue from laboratory tests and surgical procedures for both types of hospitals increased and the discrepancy between the two types of hospitals was narrowed from 1999 to 2004. For TCM hospitals, revenues from laboratory tests increased from 3.64% to 5.06% and revenues from surgical procedures increased from 3.44% to 7.02%. General hospitals' TCM drug revenue in outpatient care declined insignificantly from 5.26% to 3.87%, while the decline for the TCM hospitals was significant from 19.73% to 13.77%. The national statistics from 1999 to 2008 showed similar trends that the percentage of revenue from Western medicine for TCM hospitals increased from 59.6% in 1999 to 62.2% in 2003 and 66.1% in 2008 while the percentage of revenue from TCM for TCM hospitals decreased from 18.0% in 1999, 15.4% in 2003, and 13.7% in 2008.

**Conclusion:**

Western medicine has become a vital revenue source for TCM hospitals in the current Chinese health care environment where government subsidies to health care facilities have significantly declined. Policies need to encourage TCM hospitals to identify their own special and effective services, improve public perception, increase demand, strengthen financial sources, and ultimately make contributions to preserving one of the national treasures.

## Background

As the primary form of healthcare service in many developing countries, complementary and alternative medicine has become more popular in the world including in a number of developed countries in recent years [1]. Given the changes in disease patterns in recent decades where chronic and functional diseases, such as malignant tumor, high blood pressure, cardiovascular and cerebrovascular disease and diabetes, have become leading diseases/conditions, people began to look for solutions from alternative medical care, which has led to the rapid development and dissemination of traditional medicine [2,3]. The World Health Organization reports that 65 - 80% of the world population uses traditional medicine as their main type of healthcare service [4]. There were US$ 60 billion sales in the global herbal medicines market in 2000 [5], and by 2003, 54 countries had legalized traditional medicine (including traditional Chinese medicine) [6].

Traditional Chinese Medicine (TCM) has over 2,000 years of history in China and is well known as the quintessence of Chinese culture [7]. It has made indelible contributions to the well- being of the Chinese people [8,9]. As one type of CAM, TCM has also become an important part of health care in East Asia countries/regions, such as China, Taiwan, Hong Kong, Singapore, and Malaysia. Some TCM services such as acupuncture have been utilized in some developed countries such as Japan, Great Britain, Canada, and the United States, where it has been legalized [10].

Since 1949, the development of TCM has consistently been a health policy priority in China. In the 1950s, "Integrating TCM and Western medicine" was one of the three guidelines for health care development [11]. The government believed that the provision of adequate health services to billions of people would be impossible unless TCM was included in health care delivery [12]. In the 1980s, the central government emphasized that delivering health services through both TCM and Western medicine, as well as their integration, should be a long-term national policy. Moreover, the central government also set up quantitative indictors for characterizing TCM hospitals. County-level TCM hospitals are required to have at least 85% and 70% of their prescriptions based on either raw herbal medicines or processed herbal medicine for outpatient and inpatient services, respectively [13]. The county-level TCM hospitals are also required to have a minimum of 70% health professionals whose primary training is in TCM [14].

In the 1990s, the government issued one of the most important health care reform policies that stressed that high quality health services should be based on the integration of TCM and Western medicine [15]. TCM has experienced unprecedented rapid growth, from only four TCM hospitals nationwide with an average of 30 beds per hospital in 1950 to 2,688 TCM hospitals with a total of 350 thousand beds in 2008 [16]. For health care providers, TCM has also become an important part of their integrated medical practice [17].

However, even with a continuous policy emphasis, TCM services seem to be strongly affected by the growth of Western medicine in recent years [18,19]. Furthermore, there has been virtually no published research on the extent of the westernization of TCM services based on our literature search. This study, therefore, compares types of revenue between TCM hospitals and the Western-medicine based general hospitals in China overtime and examines what these financial trends indicate concerning the Westernization of TCM hospitals. Findings of this study serve as an evaluation of the overall effectiveness of the national policy during the last 30 years to support the growth of TCM in China, and they can be used to help develop strategies to sustain and strengthen TCM [20].

## Methods

Study design and sampling. To examine whether TCM hospitals have become more westernized over time, we hypothesized that the traditional Chinese hospitals have become more dependent on Western medical services such as laboratory tests, surgeries, as well as Western medicine. Data were obtained from two hospital surveys conducted in 1999 and 2004, respectively. We used both cross sectional and panel models to conduct the data analysis. In addition, we used the similar national statistics from 1999 to 2008 to verify consistency between our findings and the national trends.

We used the stratified randomization sampling method in 1999 for the first survey. First, we categorized the 31 Chinese provinces as Eastern, Middle, and Western regions, which generally represent well-developed, moderately-developed, and under-developed regions of China. Two provinces are drawn from each region. They are Zhejiang and Jiangsu, Jiangxi and Hunan, and Sichuan and Shaanxi in the Eastern, Middle, and Western regions, respectively. Second, in each of the six provinces, we further categorized cities into three groups based on their levels of economic development. Three cities were drawn from each level. Third, we used the same method to randomly choose three counties from all counties that are administratively affiliated with a city. Finally, we included general and TCM hospitals from each county. Hence, for each province, we collected data in three cities and nine counties. The survey stopped if there was no general hospital or TCM hospital in a selected county. As result, the final sample contained a total of 200 hospitals, 97 TCM hospitals and 103 general hospitals, and six cities/counties had more than one general hospitals. The same hospitals were followed-up in 2004 for the second survey.

Measures. During the two survey periods, data for bed size and various types of revenues were abstracted. Six response variables were identified to measure types of revenue from the different services. They were: 1) the percentage of total service revenue from laboratory tests, 2) the percentage of total service revenue from surgeries, 3) percentage of total service revenue from TCM prescriptions in outpatient services, 4) percentage of total service revenues from TCM prescriptions in inpatient services, 5) percentage of total service revenue from the Western medicine prescriptions in outpatient services, and 6) percentage of total service revenue from the Western medicine prescriptions in inpatient services. The two main independent variables were hospital type (a value of "1" for TCM versus a value of"0" for general hospital) and year (a value of "1" for the year of 2004 versus a value of "0" for the year of 1999).

Data analysis. We applied both a pooled cross sectional fixed effect model and a panel design, random effect model in investigating the relationship between hospital type and the six dependent variables over time, respectively. The panel design fixed model was not utilized, because there was no change in hospital type over time [21]. The cross sectional fixed model was able to take into account unmeasured between-hospital variation, while the panel random effect method took into account the dependence of the repeated measures of the hospitals during the two surveys. By conducting the multivariable analysis using two different but complementary methods, we were able to check the consistency of our findings.

Several hospital characteristics were controlled for in the multivariable analysis that was conducted using the STATA Version 11 software. The hospital characteristics included the number of staffed beds (in 100 s), level of hospital (i.e., city or county hospital), and region where the hospital was located. As a common practice, China categorizes its hospitals by three levels. The province-level hospital is the largest in size and most advanced, the county-level hospital is the smallest in size and least advanced, and the city-level hospital is in-between. All of the survey hospitals were public ones.

## Results

Unadjusted descriptive statistics of characteristics TCM hospitals and other hospitals are shown in Table 1. The top panel of Table 1 displays the results of the 200 hospitals we surveyed. From 1999 to 2004, both TCM hospitals and general hospitals grew in bed size and total revenues. The average bed sizes were 173 beds for the TCM hospitals and 413 beds for the general hospitals in 1999, whereas the numbers of beds were 210 and 467 for the TCM and general hospitals, respectively, in 2004. The general hospitals were sizably larger than TCM hospitals in terms of the number of staffed beds and total revenues. In 2004, the average bed size of traditional Chinese hospitals was only 45% that of general hospitals and the total revenue was only 36%. The percentages of government subsidies as a percentage of total revenue dropped from 7.2% to 4.7% for the TCM hospitals and from 4.8% to 3.4% for the general hospitals between 1999 and 2004.

**Table 1:** Characteristics of Hospitals: A Sample of 200 Hospitals and the National Average

	TCM Hospital			General Hospital		
		
200 Hospitals	1999	2004	Difference*	1999	2004	Difference*
Total Number of Hospitals	97	97	0	103	103	0
Number of staffed beds, mean (SD)	176	210	34	413	467	54
Number of TCM providers, mean (SD)	62 (49)	51 (39)	-9	13 (10)	12 (9)	-1
Mean percentage of TCM providers (SD)	52.1% (24.0%)	47.5% (21.4%)	-4.7%	8.2% (8.9%)	7.4% (7.9%)	-0.8%
Level of hospital						
Province	5.2%	5.2%	0.0%	5.8%	5.8%	0.0%
City	50.5%	49.5%	-1.0%	53.4%	53.4%	0.0%
County	44.3%	45.4%	1.0%	40.8%	40.8%	0.0%
Region of hospital located						
Eastern	34.0%	34.0%	0.0%	32.0%	32.0%	0.0%
Middle	33.0%	33.0%	0.0%	34.0%	34.0%	0.0%
Western	33.0%	33.0%	0.0%	34.0%	34.0%	0.0%
Total Revenues, in million, mean (SD)**	23.38 (37.69)	39.39 (57.10)	16.01	57.35 (59.16)	106.93 (118.89)	49.58
Total service revenues, in million, mean (SD)**	21.69 (35.64)	37.31 (52.34)	15.62	54.50 (57.12)	104.47 (112.23)	49.97
Government subsidy as percent of total revenue	7.2%	4.7%	-2.5%	4.8%	3.4%	-1.5%

	**TCM Hospital**			Other Hospital****		
		
National Statistics***	**1999**	**2003**	**2008**	**1999**	**2003**	**2008**

Number of Hospitals	2,555	2,446	2,437	6,259	5,868	6,447
Number of staffed beds, mean	104	121	150	213	239	269
Number of health care providers, mean	158	173	207	311	337	374
Level of hospital						
- Province and city	24.2%	25.2%	24.6%	44.6%	44.4%	43.0%
- County	75.8%	74.8%	75.4%	55.4%	55.6%	57.0%
Total revenues, in million yuan, mean	8.2	14.4	33.2	22.5	39.8	82.4
Government subsidy as percent of total revenue	9.5%	9.3%	10.6%	7.7%	8.3%	8.5%

SD - standard deviation

* Difference is obtained by subtracting 1999 data from 2004 data

** In million yuan RMB (Ren-Min-Bi): Chinese currency; Total revenue = Total service revenue + Government subsidy

*** Data sources: the 1999 - 2008 National Health Care Accounting Statistics, Department of Planning and Budget, Ministry of Health

**** Including general hospital, TCM-Western integration hospital, other ethnic hospital, special hospital, and nursing home; but not including prevention clinics, maternal and child health institute, and rehabilitation facilities.

The bottom panel of Table 1 shows the national statistics of TCM hospitals and other hospitals from 1999 to 2008. Both TCM hospitals and other types of hospitals had grown in bed size and other hospitals were sizably larger than TCM hospitals. The percentage of government subsidies slightly increased for both TCM hospitals (9.5%, 9.3%, and 10.6% for 1999, 2003, and 2008, respectively) and other hospitals (7.7%, 8.3%, and 8.5% for 1999, 2003, and 2008, respectively).

Table 2 shows the revenue sources of TCM hospitals and other hospitals. The top panel of Table lists the results of the 200 hospitals. More than half of the service revenues came from outpatient services for the TCM hospitals, while outpatient service revenues were less than half for the general hospitals. The percentage of revenue from Western medicine for the TCM hospitals increased (44.3% and 47.4% for 1999 and 2004, respectively) and the percentage of revenue from TCM for the TCM hospitals decreased (26.4% and 18.8% for 1999 and 2004, respectively). Similarly, the percentages of revenues generated from laboratory tests and surgeries increased for both types of hospitals over time. Laboratory revenue as a percent of total service revenue for the TCM hospitals increased from 4.1% in 1999 to 5.7% in 2004 while surgical revenue as a percent of total service revenue increased from 3.4% in 1999 to 6.9% in 2004. It is surprising that the TCM hospitals had a higher percentage of revenues from Western medicine prescriptions in outpatient care (20.1% in 1999 and 15.6% in 2004) than their general hospital counterparts (17.3% in 1999 and 14.5% in 2004). The percentage of TCM drug revenue as a percentage of total drug revenue dropped from 41.2% to 34.2% for the TCM hospitals and from 14.4% to 10.4% for the general hospitals between 1999 to 2004, and more decline was observed in outpatient care versus inpatient care.

**Table 2:** Revenue Components by Hospital Type

	TCM Hospital			General Hospital		
		
200 Hospitals	1999	2004	Difference*	1999	2004	Difference*
Outpatient revenue as a percent of total service revenue	58.0%	50.8%	-7.2%	39.0%	35.3%	-3.7%
Inpatient revenue as a percent of total service revenue	42.0%	49.2%	7.2%	61.0%	64.7%	3.7%
						
Percent of service revenue from Western medicine**	44.3%	47.4%	3.1%	55.3%	56.4%	1.1%
Percent of service revenue from TCM***	26.4%	18.8%	-7.6%	7.5%	4.9%	-2.6%
Percent of service revenue from Western medicine or TCM****	29.3%	33.8%	4.5%	37.2%	38.7%	1.5%
						
Laboratory revenue as a percent of total service revenue	4.1%	5.7%	1.6%	5.8%	7.2%	1.4%
Surgery revenue as a percent of total service revenue	3.4%	6.9%	3.5%	5.0%	7.3%	2.3%
Drug revenue as a percent of total service revenue	63.2%	53.6%	-9.6%	52.0%	46.8%	-5.2%
Western drug revenue as a percent of total service revenue	36.8%	34.8%	-2.0%	44.5%	41.9%	-2.5%
Outpatient western drug revenue as a percent of total service revenue	20.1%	15.6%	-4.5%	17.3%	14.5%	-2.8%
Inpatient western drug revenue as a percent of total service revenue	16.8%	19.2%	2.4%	27.1%	28.1%	0.9%
TCM drug revenue as a percent of total service revenue	26.4%	18.8%	-7.6%	7.5%	4.9%	-2.6%
Outpatient TCM drug revenue as a percent of total service revenue	21.0%	14.8%	-6.2%	5.3%	3.7%	-1.6%
Inpatient TCM drug revenue as a percent of total service revenue	5.3%	3.9%	-1.4%	2.2%	1.3%	-0.9%
						
TCM drug revenue as a percent of total drug revenue	41.2%	34.2%	-7.0%	14.4%	10.4%	-4.0%
Outpatient TCM drug revenue as a percent of total drug revenue	32.4%	27.0%	-5.4%	10.0%	7.7%	-2.3%
Inpatient TCM drug revenue as a percent of total drug revenue	8.8%	7.3%	-1.5%	4.4%	2.9%	-1.5%
Outpatient TCM drug revenue as a percent of total outpatient drug revenue	50.4%	47.5%	-2.9%	23.6%	19.9%	-3.7%
Inpatient TCM drug revenue as a percent of total inpatient drug revenue	24.7%	16.8%	-7.9%	7.6%	4.6%	-3.0%

	**TCM Hospital**			Other Hospital******		
		
National Statistics*****	**1999**	**2003**	**2008**	**1999**	**2003**	**2008**

Outpatient revenue as a percent of total service revenue	58.8%	52.5%	46.1%	43.2%	39.5%	44.5%
Inpatient revenue as a percent of total service revenue	41.2%	47.5%	53.9%	56.8%	60.5%	55.7%
	0.0%	0.0%	0.0%	0.0%	0.0%	0.0%
Percent of service revenue from Western medicine**	59.6%	62.2%	66.1%	82.9%	88.3%	92.1%
Percent of service revenue from TCM***	18.0%	15.4%	13.7%	3.4%	1.9%	2.6%
Percent of service revenue from Western medicine or TCM****	22.4%	22.4%	20.3%	13.8%	9.8%	31.0%
						
Laboratory revenue as a percent of total service revenue	7.6%	8.8%	10.4%	9.4%	10.3%	11.6%
Surgery revenue as a percent of total service revenue	3.4%	4.9%	5.7%	4.6%	6.4%	7.0%
Drug revenue as a percent of total service revenue	61.2%	55.0%	51.7%	52.6%	48.0%	46.1%
Western drug revenue as a percent of total service revenue	36.5%	34.2%	34.2%	43.6%	42.4%	42.4%
Outpatient western drug revenue as a percent of total service revenue	20.3%	16.0%	13.6%	19.6%	17.0%	15.8%
Inpatient western drug revenue as a percent of total service revenue	16.2%	18.2%	20.5%	24.0%	25.4%	26.6%
TCM drug revenue as a percent of total service revenue	11.0%	8.5%	7.1%	1.8%	0.9%	1.2%
Outpatient TCM drug revenue as a percent of total service revenue	9.4%	7.5%	6.2%	1.4%	0.8%	1.0%
Inpatient TCM drug revenue as a percent of total service revenue	1.6%	1.0%	0.9%	0.4%	0.1%	0.3%
	0.0%	0.0%	0.0%	0.0%	0.0%	0.0%
TCM drug revenue as a percent of total drug revenue	18.0%	15.4%	13.7%	3.4%	1.9%	2.6%
Outpatient TCM drug revenue as a percent of total drug revenue	15.3%	13.6%	11.9%	2.7%	1.6%	2.1%
Inpatient TCM drug revenue as a percent of total drug revenue	2.6%	1.8%	1.8%	0.7%	0.3%	0.6%
Outpatient TCM drug revenue as a percent of total outpatient drug revenue	23.4%	22.8%	22.7%	5.3%	3.6%	3.6%
Inpatient TCM drug revenue as a percent of total inpatient drug revenue	7.7%	4.4%	3.7%	1.4%	0.6%	1.3%

* Difference is obtained by subtracting 1999 data from 2004 data

** Including lab tests, surgeries, and Western medications.

*** Including TCM medications.

**** Including registration, physician services, and diagnoses (e.g., CT scanner, MRI, ultrasound) but cannot be distinguished between TCM and Western medicine.

***** Data sources: the 1999 - 2008 National Health Care Accounting Statistics, Department of Planning and Budget, Ministry of Health

****** Including general hospital, TCM-Western integration hospital, other ethnic hospital, special hospital, and nursing home; but not including prevention clinics, maternal and child health institute, and rehabilitation facilities.

The national statistics from 1999 to 2008 show similar trends (the bottom panel of Table 2). The percentage of revenue from Western medicine for TCM hospitals increased (59.6% in 1999, 62.2% in 2003, and 66.1% in 2008) and the percentage of revenue from TCM for TCM hospitals decreased (18.0% in 1999, 15.4% in 2003, and 13.7% in 2008). Specifically, laboratory revenue as a percent of total service revenue were 7.6%, 8.8%, and 10.4% in 1999, 2003, and 2008, respectively, and surgical revenues as a percent of total service revenue were 3.4%, 4.9%, and 5.7% in 1999, 2003, and 2008, respectively. Furthermore, TCM drug revenue as a percent of total drug revenue declined from 18.0% in 1999 to 15.4% in 2003, and 13.7% in 2008.

Results of the multivariable analysis of the cross sectional models are shown in Table 3. For the six dependent variables, compared with general hospitals, TCM hospitals had lower percentages of revenues for three types of revenue, a comparable percentage for one type, and higher percentages for two types. Specifically, their percentages of revenues from laboratory tests, surgical procedures, and inpatient Western drug prescriptions were 1.01%, 1.96%, and 8.94% lower than those of their general hospital counterparts, and their percentage of revenue for outpatient care Western medicine was comparable to their general hospital counterparts in 1999. Moreover, for the general hospitals, the percentages of revenue from laboratory tests and surgical procedures increased by 1.24% and 2.43% from 1999 to 2004, respectively, but the percentages of revenue from outpatient Western drug prescriptions decreased by 3.98% from 1999 to 2004. Since none of the interaction terms between TCM and time was statistically significant, the differences between the two types of hospitals did not change significantly over time, meaning that for the TCM hospitals, from 1999 to 2004, the percentages of revenue from laboratory tests and surgical procedures increased by 1.32% and 3.58%, respectively, and the percentages of revenue from outpatient Western drug prescriptions decreased by 3.98%.

**Table 3:** Results of the Pooled Cross Sectional Analysis (n = 368)

Dependent Variable	Independent Variable	**Coef**.	**95% C.I**.	P-Value	R-square
Lab test revenue as a percent of total revenue					0.33
	constant	4.65%	(4.05, 5.23)	<.001	
	type = TCM	-1.01%	(-1.61, -0.41)	0.001	
	year = 2004	1.24%	(0.72, 1.77)	<.001	
	type*year	0.18%		0.636	
					
Surgical revenue as a percent of total revenue					0.18
	constant	5.40%	(4.25, 6.55)	<.001	
	type = TCM	-1.96%	(-3.12, -0.80)	0.001	
	year = 2004	2.43%	(1.41, 3.46)	<.001	
	type*year	1.15%		0.121	
					
TCM drug revenue as a percent of total revenue for outpatient care					0.48
	constant	5.26%	(2.76, 7.75)	<.001	
	type = TCM	14.47%	(11.95, 16.98)	<.001	
	year = 2004	-1.39%		0.219	
	type*year	-4.57%	(-7.72, -1.41)	0.005	
					
TCM drug revenue as a percent of total revenue for inpatient care					0.27
	constant	2.32%	(1.35, 3.29)	<.001	
	type = TCM	2.75%	(1.77, 3.73)	<.001	
	year = 2004	-0.82%	(-1.68, 0.04)	0.062	
	type*year	-0.65%		0.299	
					
Western drug revenue as a percent of total revenue for outpatient care					0.26
	constant	18.59%	(16.56, 20.62)	<.001	
	type = TCM	1.21%		0.169	
	year = 2004	-3.98%	(-5.33, -2.64)	<.001	
					
Western drug revenue as a percent of total revenue for inpatient care					0.40
	constant	23.92%	(21.87, 25.97)	<.001	
	type = TCM	-8.94%	(-11.00, -6.88)	<.001	
	year = 2004	0.45%		0.629	
	type*year	1.76%		0.18	

Coef. - regression coefficients; 95 C.I. - 95% confidence interval for regression coefficient.

The model controlled covariates including the number of staffed beds, hospital level, and region of hospital located.

Understandably, the two higher percentages of drug revenues for the TCM hospitals were from TCM prescription drugs in outpatient and inpatient care, respectively. While the discrepancy in inpatient care between the two types of hospitals remained unchanged over time, the discrepancy in outpatient care significantly narrowed. For the general hospitals, the TCM drug revenue as a percentage of total service revenues for outpatient care declined insignificantly from 5.26% to 3.87% from 1999 to 2004, while there was a significant decline for the TCM hospitals from 19.73% to 13.77% during the same period.

Results from the multivariable panel model, as shown in Table 4, were generally consistent to those findings in Table 3. There was one more statistically significant interaction in the percentage of revenue from surgical procedures, indicating that the discrepancies in the percentage of revenues from surgical procedures between the two types of hospitals narrowed by 1.24% from 1999 to 2004. Specifically, revenues for surgical procedures increased from 5.51% to 7.91% for the general hospitals between 1999 and 2004, but they more than doubled from 3.43% to 7.07%, for TCM hospitals during the same period.

**Table 4:** Results of the Random Effect Panel Design Model (n = 193)

Dependent Variable	Independent Variable	**Coef**.	**95% C.I**.	p-Value	R-square
Lab test revenue as a percent of total revenue					0.33
	constant	4.69%	(4.06, 5.33)	<.001	
	type = TCM	-1.05%	(-1.66, -0.45)	0.001	
	year = 2004	1.26%	(0.81, 1.71)	<.001	
	type*year	0.18%		0.568	
					
Surgical revenue as a percent of total revenue					0.18
	constant	5.51%	(4.27, 6.74)	<.001	
	type = TCM	-2.08%	(-3.25, -0.90)	0.001	
	year = 2004	2.40%	(1.59, 3.22)	<.001	
	type*year	1.24%	(0.10, 2.39)	0.033	
					
TCM drug revenue as a percent of total revenue for outpatient care					0.48
	constant	5.15%	(2.37, 7.93)	<.001	
	type = TCM	14.49%	(11.94, 17.05)	<.001	
	year = 2004	-1.31%	(-2.73, 0.10)	0.069	
	type*year	-4.78%	(-6.75, -2.81)	<.001	
					
TCM drug revenue as a percent of total revenue for inpatient care					0.27
	constant	2.17%	(1.11, 3.23)	<.001	
	type = TCM	2.82%	(1.82, 3.81)	<.001	
	year = 2004	-0.85%	(-1.51, -0.19)	0.012	
	type*year	-0.55%		0.249	
					
Western drug revenue as a percent of total revenue for outpatient care					0.26
	constant	18.70%	(16.41, 20.98)	<.001	
	type = TCM	1.12%		0.273	
	year = 2004	-3.95%	(-4.97, -2.95)	<.001	
					
Western drug revenue as a percent of total revenue for inpatient care					0.40
	constant	24.15%	(21.88, 26.41)	<.001	
	type = TCM	-9.15%	(-11.26, -7.04)	<.001	
	year = 2004	0.53%		0.43	
	type*year	1.73%	(-0.11, 3.57)	0.067	

Coef. - regression coefficients; 95 C.I. - 95% confidence interval for regression coefficient.

The model controlled covariates including the number of staffed beds, hospital level, and region of hospital located.

## Discussion

Our findings indicate that the Western medicine is a vital and major financial source for the survival of TCM hospitals in China. Most TCM hospital's revenues are generated though providing services based on the Western medicine. Although we used revenue to approximate the number of prescriptions, it is obvious that TCM hospitals are far from meeting the national standard that they prescribe at least 85% and 70% of their prescriptions based on herbal medicines for the outpatient and inpatient services, respectively [14]. More undesirably, TCM hospitals' percentage of TCM medication revenue, instead of moving up towards the national standard, has gone down over time. The gradual increase in Western medicine in the TCM hospitals is leading to the demise of their traditional features and strengths, which, in turn, is resulting in the weakening of their competitive advantages over general hospitals in local markets [22].

While differences in inpatient drug prescriptions between TCM hospitals and general hospitals remained steady over time, TCM hospitals have become more similar to general hospitals in regard to outpatient prescriptions. This is particularly significant given that TCM hospitals rely more financially on outpatient care than general hospitals, since more than half of their service revenues come from outpatient services. The gap in outpatient care has been narrowed, which demonstrates yet another isomorphic behavior between the two types of hospitals. It is not to understand why TCM hospitals do not prioritize traditional medications over Western medications. This "Westernization" behavior indicates TCM hospitals' pragmatic approach in order to survive.

Both TCM and general hospitals utilized more lab tests and surgeries, which are Western medicine practices, to boost their revenues in 2004 as compared to 1999. The national data from 1999 to 2008 observed the similar increase. Moreover, the increase in TCM hospitals' percentage of surgical revenues was larger than that of general hospitals over time. This further indicates that TCM hospitals have become more "Westernized" in terms of revenue sources.

There are several explanations for why TCM hospitals are becoming more like general hospitals. First of all, demand for TCM services has declined. It is reported that 94.8% of rural residents prefer to see a Western medicine doctor first when they are sick, as compared to only 5.2% of them who choose to see a TCM doctor first [23]. Research has also suggested that 84.9% of health care providers and health care administrators prefer to seek out Western medical care first when they get sick, compared to 15.1% who would choose TCM first [23]. It appears that TCM service providers may also relegate TCM services to the second place. It seems to be that, along with the fast economic growth, modern and advanced Western medicine has become more popular and acceptable and, at the same time, the public trust to and confidence about slowly updated TCM are facing challenges in China, which negatively affects its demand. Given the fact that government subsidies have significantly shrunk and that most of hospital revenues are generated from medical services delivery, lack of demand for services threatens the financial variability of TCM hospitals. They have to rely on more popular Western medicine as their primary revenue source in order to survive.

Second, the national policy of promoting TCM, especially the "Let each county have a TCM hospital" initiative, does not seem to have achieved its policy goals. In contrast, TCM hospitals appear to downplay TCM. The original intention to build TCM hospitals for all counties was to increase the access to and availability of TCM services. However, instead of offering TCM care as their primary services, TCM health care providers and hospitals are providing more and more Western medical services. Our findings show increases in the proportions of total revenues generated by Western medicine oriented lab tests and surgeries, as well as the proportion of the total medication revenue generated from Western drugs. Given that TCM hospitals are growing more similar to general hospitals, one may raise a question about whether it is necessary to have a stand-alone TCM hospital in each county, regardless of the demand for TCM in that county. Moreover, the TCM policy failed to build well-functioning TCM hospitals with typical and strong TCM features. The "Let each county have a TCM hospital" policy was supposed to speed up the development of TCM services, but newly built TCM hospitals are becoming more like general hospitals in the services they provide.

The TCM hospital's behavior of providing less and less TCM care may be also related to its own features and characteristics which cannot keep up with Western medicine in market competition. Compared to Western medicine, TCM treatment processes are often longer and non-replicable and their treatment outcomes are more delayed. In addition, TCM is not particularly effective in dealing with acute or/and infectious diseases [14]. As a result, TCM hospitals face a dilemma in the current health care environment. Socially, it is a privilege and moral responsibility for TCM hospitals to maintain the national treasure by offering most of their services based on TCM. On the other hand, TCM hospitals must keep themselves financially healthy. To achieve that, they have no choice but to provide more Western medicine because revenues generated from TCM services are not sufficient. Studies have shown that TCM hospitals that tried to keep TCM as their primary revenue source experienced slow growth, decline of service volume, shrinking market share, and ultimately they were struggling for survival [19]. It is reported that one-third of TCM hospitals struggle financially and are in danger of being shut down, merged, or relocated [24]. It is understandable why TCM hospitals provide more and more Western medicine based care.

Several limitations existed in this research. First, we compared TCM and general hospitals in percentage of revenues for laboratory tests, surgeries, and prescription drugs. Other types of revenues, such as registration fees, physician service fees, and diagnostic fees, were not examined because the information could not be distinguished between TCM and Western health care providers within hospitals. In addition, given the focus on types of revenues in this study, we did not compare health care providers and other human resource factors between TCM and general hospitals. Third, due to the inconsistency between the 1999 and 2004 surveys, revenue generated from high-tech services, such as MRI, CT, and ultrasonic, were not investigated. Fourth, due to the limited sample size, hospitals at different levels or regions were not analyzed separately. Fifth, the non-randomization in selecting the hospitals might limit the generalibilty of our findings and the data we used are seven years old. Nevertheless, the more recent national statistics from 1999 to 2008 showed the same trends of Westernization of TCM hospitals we found in the study. Finally, most of the cited literature is in Chinese, which makes it difficult for English readers to trace the original articles.

## Conclusion

Even with the strong national policy support to enhance TCM access and availability and TCM is an important part of integrated medical practice [17], TCM hospitals still tend to become more Westernized. It is important to understand why the implementation of the policies deviates from the original goals. The reality is that Western medicine is a vital revenue source for the survival of TCM hospitals, and they have become more reliant on it in the current health care environment in China where government subsidies to health care facilities have significantly declined. In order to maintain the vigorousness of traditional Chinese medicine, policies to strengthen TCM should re-emphasize their standards or requirements (e.g., for TCM hospitals, 80% TCM prescriptions for outpatient care and 70% for inpatient care) and they should allocate special funds for updating and upgrading TCM. More specifically, instead of the general support and across the board fund allocation policy that has been implemented for over three decades, policies need to support efforts of identifying a range of specific TCM services that are more effective than Western medicine in dealing with certain diseases, especially some chronic diseases and clinical conditions related to aging. Each TCM hospital needs to eventually develop its own TCM related clinical specialties. By doing so, TCM hospitals can gain comparative advantage over Western medicine hospitals, improve public perception, increase demand, strengthen financial sources, and ultimately go beyond just survival and make contributions to preserving one of the national treasures of China.

## Competing interests

The authors declare that they have no competing interests.

## Authors' contributions

JS participated in design of study, data analysis and interpretation, statistical methods, manuscript draft, administrative and technical assistance, and supervision. YW participated in design and concept of study, acquisition of data, manuscript draft, and administrative and material assistance. FL participated in data analysis and interpretation and manuscript draft. JL participated in concept of the study and acquisition of data. CM participated in data analysis and interpretation and manuscript draft. MS participated in concept of study and acquisition of data. MH participated in design and concept of study, acquisition of data, manuscript draft, acquisition of funding, and supervision. All authors read and approved the final manuscript.

## Pre-publication history

The pre-publication history for this paper can be accessed here:

http://www.biomedcentral.com/1472-6963/11/212/prepub
